# Schistosomiasis in school-age children in Burkina Faso after a decade of preventive chemotherapy

**DOI:** 10.2471/BLT.15.161885

**Published:** 2015-11-24

**Authors:** Hamado Ouedraogo, François Drabo, Dramane Zongo, Mohamed Bagayan, Issouf Bamba, Tiba Pima, Fanny Yago-Wienne, Emily Toubali, Yaobi Zhang

**Affiliations:** a Programme National de Lutte contre la Schistosomiase, Ministère de la Sante, Ouagadougou, Burkina Faso.; b Coordination des Maladies Tropicales Négligées, Ministère de la Sante, Ouagadougou, Burkina Faso.; c Institut de Recherche en Sciences de la Santé, Ouagadougou, Burkina Faso.; d Helen Keller International, Ouagadougou, Burkina Faso.; e Helen Keller International, New York, United States of America.; f Helen Keller International, Regional Office for Africa, BP 29.898, Dakar-Yoff, Senegal.

## Abstract

**Objective:**

To assess the impact of a decade of biennial mass administration of praziquantel on schistosomiasis in school-age children in Burkina Faso.

**Methods:**

In 2013, in a national assessment based on 22 sentinel sites, 3514 school children aged 7–11 years were checked for *Schistosoma haematobium* and *Schistosoma mansoni* infection by the examination of urine and stool samples, respectively. We analysed the observed prevalence and intensity of infections and compared these with the relevant results of earlier surveys in Burkina Faso.

**Findings:**

*S. haematobium* was detected in 287/3514 school children (adjusted prevalence: 8.76%, range across sentinel sites: 0.0–56.3%; median: 2.5%). The prevalence of *S. haematobium* infection was higher in the children from the Centre-Est, Est and Sahel regions than in those from Burkina Faso’s other eight regions with sentinel sites (*P* < 0.001). The adjusted arithmetic mean intensity of *S. haematobium* infection, among all children, was 6.0 eggs per 10 ml urine. Less than 1% of the children in six regions had heavy *S. haematobium* infections – i.e. at least 50 eggs per 10 ml urine – but such infections were detected in 8.75% (28/320) and 11.56% (37/320) of the children from the Centre-Est and Sahel regions, respectively. *Schistosoma mansoni* was only detected in two regions and 43 children – i.e. 1 (0.31%) of the 320 from Centre-Sud and 42 (8.75%) of the 480 from Hauts Bassins.

**Conclusion:**

By mass use of preventive chemotherapy, Burkina Faso may have eliminated schistosomiasis as a public health problem in eight regions and controlled schistosome-related morbidity in another three regions.

## Résumé

**Objectif:**

Évaluer l'impact d'une décennie de distribution biennale massive de praziquantel sur la schistosomiase chez les enfants d'âge scolaire au Burkina Faso.

**Méthodes:**

En 2013, dans le cadre d'une évaluation nationale basée sur 22 postes-sentinelles, un examen des urines et des échantillons des selles ont respectivement été utilisés pour déceler des infections à la *Schistosoma haematobium* et à la *Schistosoma mansoni* chez 3514 écoliers âgés de 7 à 11 ans. Nous avons analysé la prévalence et l'intensité des infections, puis nous les avons comparées avec les résultats pertinents de précédentes études réalisées au Burkina Faso.

**Résultats:**

La *Schistosoma haematobium* a été détectée chez 287 des 3514 écoliers (prévalence ajustée: 8,76%; étendue entre les postes-sentinelles: 0,0–56,3%; moyenne: 2,5%). La prévalence de l'infection à la *Schistosoma haematobium* était plus élevée chez les enfants des régions Centre-Est, Est et du Sahel que chez les enfants des huit autres régions du Burkina Faso comprenant des postes-sentinelles (*P* < 0,001). La moyenne arithmétique ajustée de l'intensité de l'infection à la *Schistosoma haematobium* était, chez tous les enfants, de 6,0 œufs pour 10 ml d'urine. Moins d'1% des enfants dans six régions souffraient d'infections graves à la *Schistosoma haematobium* – au moins 50 œufs pour 10 ml d'urine –, mais de telles infections ont été détectées chez respectivement 8,75% (28/320) et 11,56% (37/320) des enfants des régions Centre-Est et du Sahel. La *Schistosoma mansoni* a seulement été détectée dans deux régions et chez 43 enfants – à savoir 1 (0,31%) des 320 enfants de la région Centre-Sud et 42 (8,75%) des 480 enfants de la région des Hauts Bassins.

**Conclusion:**

Grâce à un recours massif à la chimiothérapie préventive, le Burkina Faso a peut-être éradiqué la schistosomiase en tant que problème de santé publique dans huit régions et endigué la morbidité liée à la schistosomiase dans trois autres régions.

## Resumen

**Objetivo:**

Evaluar el efecto de una década de administración bienal de praziquantel en masa para la esquistosomiasis en los niños en edad escolar de Burkina Faso.

**Métodos:**

En 2013, en una evaluación nacional basada en 22 sitios centinela, se examinó a 3 514 alumnos de entre 7 y 11 años para saber si estaban infectados por *Schistosoma haematobium* y *Schistosoma mansoni* mediante un examen de muestras de orina y heces, respectivamente. Se analizó la prevalencia e intensidad observadas de las infecciones y se compararon con los resultados relevantes de exámenes anteriores en Burkina Faso.

**Resultados:**

Se encontró *S*. *haematobium *en 287/3 514 alumnos (prevalencia ajustada: 8,76%, rango entre sitios centinela: 0,0–56,3%; mediana: 2,5%). La prevalencia de la infección por *S. haematobium* era mayor en los niños de las regiones Centro-Este, Este y del Sahel que en los niños de las otras ocho regiones con sitios centinela de Burkina Faso (*P* < 0,001). La intensidad aritmética ajustada media de la infección por *S. haematobium*, entre todos los niños, era de 6,0 huevos por cada 10 ml de orina. Menos del 1% de los niños en seis regiones sufría de infecciones fuertes por *S. haematobium* (es decir, al menos 50 huevos por 10 ml de orina), pero se detectaron tales infecciones en un 8,75% (28/320) y un 11,56% (37/320) de los niños del Centro-Este y de las regiones del Sahel, respectivamente. Solo se detectó *Schistosoma mansoni* en dos regiones y 43 niños, es decir, 1 (0,31%) de los 320 del Centro-Sur y 42 (8,75%) de los 480 de las altas cuencas.

**Conclusión:**

Gracias al uso masivo de quimioterapia preventiva, Burkina Faso puede haber eliminado la esquistosomiasis como un problema de salud pública en ocho regiones y controlado la morbilidad relacionada con la esquistosomiasis en otras tres regiones.

## ملخص

**الغرض:**

تقييم تأثير توزيع دواء "البرازيكوانتيل" على نطاق عمومي كل سنتين على مدار 10 سنوات في داء البلهارسيا المصاب به الأطفال من سن الدراسة بدولة بوركينا فاصو.

**الطريقة:**

في عام 2013، جرى تقييم وطني مستند إلى 22 موقعًا خفريًا، وتم فحص 3514 طفلاً من أطفال المدارس الذين تتراوح أعمارهم من 7 إلى 11 عامًا للكشف عن عدوى *البلهارسيا الدموية * و *البلهارسيا المنسونية * من خلال تحليل عينات البول والبراز، على التوالي. وقمنا بتحليل معدل الانتشار المراقب وشدة العدوى، كما قمنا بمقارنة هذه التحاليل بالنتائج ذات الصلة للمسوح السابقة في بوركينا فاصو.

**النتائج:**

تم اكتشاف عدوى *البلهارسيا الدموية * في 287/3514 من أطفال المدارس (معدل الانتشار المصحح: 8.76%، عبر نطاق المواقع الخفرية: 0.0–56.3%؛ المتوسط: 2.5%). كان معدل انتشار عدوى *البلهارسيا الدموية * أكبر بين الأطفال المقيمين في المناطق الوسطى من شرق البلاد، والمناطق الشرقية، ومنطقة الساحل عن أولئك الأطفال المقيمين في ثماني مناطق أخرى في بوركينا فاصو مع مواقع خفرية ( *الاحتمال * < 0.001). إن المتوسط الحسابي المصحح يعني أن معدل شدة عدوى *البلهارسيا الدموية *، من بين جميع الأطفال، كان 6.0 بيضة في كل 10 مل من البول. كانت نسبة الأطفال المصابين بعدوى شديدة من *البلهارسيا الدموية * أقل من 1% في ست مناطق – أي وجود ما لا يقل عن 50 بيضة في كل 10 مل من البول – ولكن تم اكتشاف مثل حالات العدوى هذه بنسبة 8.75% (28/320) و11.56% (37/320) في الأطفال المقيمين في المناطق الوسطى من شرق البلاد والساحل، على التوالي. تم اكتشاف عدوى " *البلهارسيا المنسونية" * في منطقتين كما بلغ قوام المصابين بها 43 طفلاً فقط – أي بواقع طفل واحد (بنسبة 0.31%) من بين 320 طفلاً من المنطقة الوسطى بجنوب البلاد، و42 طفلاً (بنسبة 8.75%) من بين 480 طفلاً من منطقة "أو باسان".

**الاستنتاج:**

لعل النجاح كان حليف بوركينا فاصو في القضاء على داء البلهارسيا كمشكلة صحية عامة في ثماني مناطق عبر استخدام العلاج الكيميائي الوقائي على نطاق عمومي، كما نجحت في ضبط معدل الإصابة بالأمراض المتصلة بعدوى البلهارسيا في ثلاث مناطق أخرى.

## 摘要

**目的:**

旨在评估为期十年（两年一次）大规模吡喹酮施用对布基纳法索学龄儿童血吸虫病的影响。

**方法:**

2013 年开展的全国性评估活动对 22 个前哨地区 3514 名 7 至 11 岁学龄儿童分别进行了尿液和粪便采样化验，以检测埃及裂体吸虫和曼氏裂体吸虫感染情况。我们对活动观察到的感染率和感染强度进行了分析，并将其与布基纳法索早期相关调查结果进行比较。

**结果:**

检测发现学龄儿童中埃及裂体吸虫感染比例为 287/3514（调整后感染率为：8.76%；前哨地区感染率范围为：0.0-56.3%；中值为：2.5%)。中东部、东部和萨赫勒大区儿童埃及裂体吸虫感染率高于布基纳法索其他八个前哨地区 (*P* < 0.001)。调整后数据表明埃及裂体吸虫在所有儿童中的感染强度为每 10 ml 尿液中有 6.0 个虫卵。六个地区儿童中患有重度（即，每 10 ml 尿液中有 50 个虫卵）埃及裂体吸虫感染的不足 1%，检测发现，中东部和萨赫勒大区此感染率分别为 8.75% (28/320) 和 11.56% (37/320)。仅在两个地区的 43 名儿童中检测到曼氏裂体吸虫，其中中南大区 1 名（共 320 名儿童），感染率为：0.31%；上盆地大区 42 名（共 480 名），感染率为：8.75%。

**结论:**

通过大规模施用化学预防疗法，布基纳法索可在八个地区清除血吸虫病这一危害公众健康的问题；同时可在另外三个地区控制与血吸虫有关的疾病感染率。

## Резюме

**Цель:**

Оценить влияние массового применения празиквантела, повторяющегося каждые два года в течение десяти лет, на распространенность шистомосоза у детей школьного возраста в Буркина-Фасо.

**Методы:**

В 2013 году в ходе национальной оценки, проводимой на 22 постах наблюдения, 3514 школьников в возрасте от 7 до 11 лет были проверены на наличие инфекций *Schistosoma haematobium* и *Schistosoma mansoni* путем анализа образцов мочи и кала соответственно. Была проанализирована наблюдаемая распространенность и степень тяжести инфекций, после чего эти показатели были сопоставлены с соответствующими результатами предыдущих исследований в Буркина-Фасо.

**Результаты:**

*S. haematobium* была обнаружена у 287 из 3514 школьников (скорректированная распространенность: 8,76%, разброс среди пунктов наблюдения: 0,0–56,3%; средняя величина: 2,5%). Распространенность инфекции *S. haematobium* у детей из Восточно-Центральной и Восточной областей и из Сахеля была выше, чем у детей из остальных восьми областей Буркина-Фасо, в которых расположены пункты наблюдения (*P* < 0,001). Скорректированное среднее арифметическое значение тяжести инфекции *S. haematobium* среди всех детей составило 6,0 яйца на 10 мл мочи. Менее чем у 1% детей в шести областях степень тяжести *S. haematobium* была высокой, т. е. по меньшей мере 50 яиц на 10 мл мочи, но инфекции такой степени тяжести были обнаружены у 8,75% (28 из 320) и 11,56% (37 из 320) детей из Восточно-Центральной области и Сахеля соответственно. *Schistosoma mansoni* была обнаружена только в двух областях и у 43 детей, т. е. у 1 (0,31%) из 320 детей из Южно-Центральной области и у 42 (8,75%) из 480 детей из области Верхние Бассейны.

**Вывод:**

Благодаря массовому применению профилактической химиотерапии в Буркина-Фасо такую проблему здравоохранения, как шистосомоз, можно считать решенной в восьми областях, а в других трех областях осуществляется контроль за заболеваемостью, связанной с шистосомой.

## Introduction

Human schistosomiasis is endemic in 78 countries or territories.^1^^,^^2^ It has been estimated that, in 2013, there were nearly 261 million people – including about 240 million in Africa – who required preventive chemotherapy because they were at risk of schistosome infection.^1^ Following the 2001 World Health Assembly resolution WHA54.19,^3^ several endemic countries in Africa launched national programmes for the control of schistosomiasis.^4^^,^^5^ These programmes are largely based on preventive chemotherapy with praziquantel and are targeted at school-age children and adults at risk.^6^ In resolution WHA65.21, the World Health Assembly called on all countries with endemic schistosomiasis to intensify their control programmes and, where appropriate, to initiate campaigns for the elimination of schistosomiasis.^7^

**Fig. 1:**
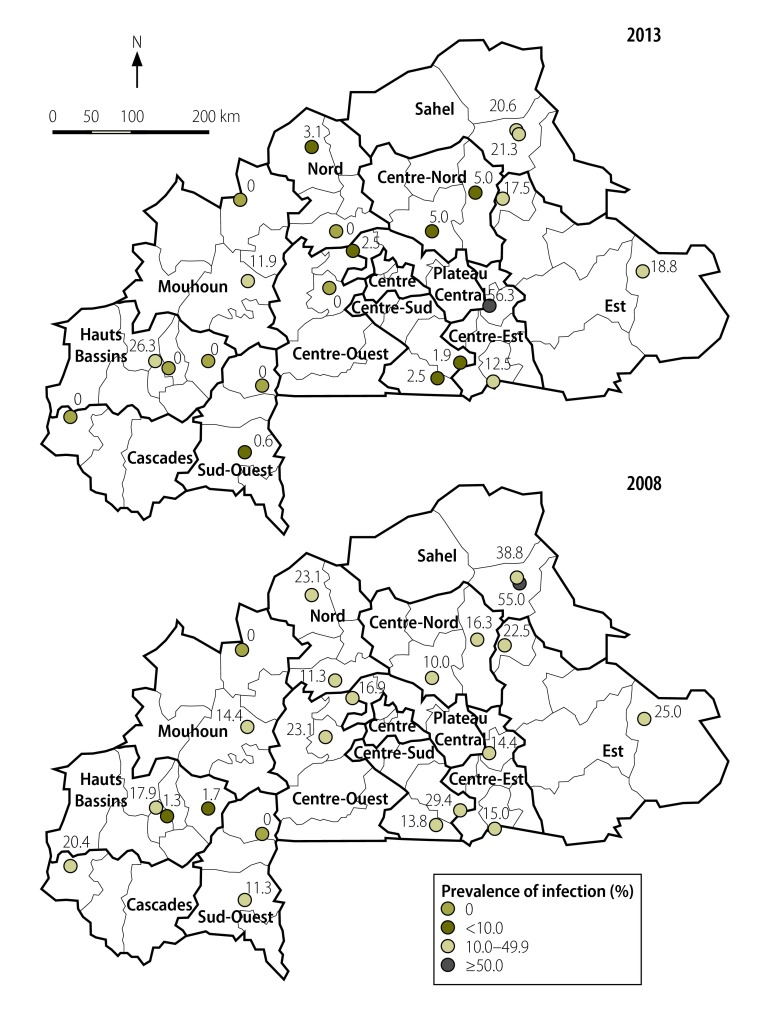
Prevalence of *Schistosoma haematobium* infection among children aged 7–11 years in 22 sentinel sites, Burkina Faso, 2008 and 2013

Notes: Each map shows the country’s 13 regions subdivided into 63 health districts. Each coloured circle indicates the approximate location of a sentinel site and the prevalence of infection recorded at that site. The values shown for 2008 were previously published by the Ministry of Health.^8^

Source: Map drawn in ArcMap version 10 (ESRI, Redlands, USA).

The West African country of Burkina Faso is divided into 13 administrative regions (Fig. 1). Some form of human schistosomiasis is thought to be endemic in every one of the country’s 63 health districts.^9^^–^^11^ Although urogenital schistosomiasis – caused by *Schistosoma haematobium* – occurs throughout the country, intestinal schistosomiasis – caused by *Schistisoma mansoni* – is mainly confined to the south-west of the country.^9^^,^^11^ Surveys conducted before the 1980s, showed that the prevalence of *S. haematobium* was very high, with focal prevalence up to 100% of people surveyed in the eastern part of the country.^9^ Over the same period, *S. mansoni* infection was found in up to 79% of people surveyed in the Hauts Bassins and Sud-Ouest regions.^9^

Burkina Faso established a national programme for the control of schistosomiasis and soil-transmitted helminths in 2004, with funding from the Schistosomiasis Control Initiative.^5^^,^^12^^,^^13^ This programme’s main objective was to use mass administration of praziquantel to prevent human schistosomiasis. National mapping surveys^14^ led to the country being divided into a hyper-endemic zone – comprising the 19 health districts that make up the Boucle du Mouhoun, Nord, Sahel and Sud-Ouest regions – and a meso-endemic zone – comprising the country’s other 44 health districts. In 2004, baseline data were collected from children attending 16 randomly-selected primary schools in the four regions of the hyper-endemic zone. Depending on the study region, the observed prevalence of *S. haematobium* infection varied from 18.4% to 84.2% and the observed intensity of such infection – among all children investigated – varied from 39.4–126.9 eggs per 10 ml urine sample.^15^ Biennial mass administration of praziquantel to school-age children began in the hyper-endemic zone in 2004 and in the meso-endemic zone in 2005.^13^^,^^15^ Since 2006, adults who are considered to be at risk have also been targeted.

In 2007, Burkina Faso’s national programme for the control of schistosomiasis and soil-transmitted helminths became part of a national integrated programme against neglected tropical diseases. The integrated programme was initially supported by the Schistosomiasis Control Initiative and *Réseau International Schistosomiases – Environnement Aménagements et Lutte*, with funding from the United States Agency for International Development’s (USAID) Neglected Tropical Disease Control Programme, managed by RTI International.^16^ Since 2011, the programme has been supported by Helen Keller International, with funding from the USAID’s End Neglected Tropical Diseases in Africa Project, managed by Family Health International 360.

At the beginning of 2013, four and five rounds of mass praziquantel administration were done in the meso-endemic and hyper-endemic zones, respectively. To assess the impact of these rounds and plan for the next phase, primary-school children at 22 sentinel sites were tested for schistosomiasis in 2013. Here we present the results of the assessment and discusses possible future strategies for the elimination of all forms of schistosomiasis from Burkina Faso.

## Methods

### Ethical considerations

The assessment survey formed part of the monitoring and evaluation activities of the programme. It was conducted by the national monitoring and evaluation team and was authorized by the Ethics Committee of the Ministry of Health of Burkina Faso. Before the survey, written informed consent was obtained from the head teacher of each study school and verbal informed consent was obtained from a parent or guardian of each child. Each child was given a unique identification number so that data could be analysed anonymously.

### Mass drug administration

Although the national strategy included biennial praziquantel rounds, the amalgamation of the national programme for schistosome control into the integrated programme for the control of neglected tropical diseases led to some scheduled administrations being missed (Table 1).

**Table 1:** Coverage of mass praziquantel administrations among school-age children, Burkina Faso, 2004–2013

Region	District	Estimated coverage (% of eligible children)^a^									
		2004	2005	2006	2007	2008	2009	2010	2011	2012	2013
Boucle du Mouhoun	Dedougou	79.92	–	92.00	–	–	83.45	–	91.00	–	97.69
	Boromo	96.35	–	89.19	–	–	92.06	–	90.81	–	101.43
	Nouna	104.34	–	94.01	–	–	91.37	–	86.58	–	91.44
	Solenzo	89.37	–	90.25	–	–	88.28	–	88.41	–	94.27
	Tougan	97.74	–	94.08	–	–	89.91	–	89.35	–	98.06
	Toma	97.37	–	96.35	–	–	94.58	–	93.09	–	94.25
Cascades	Banfora	–	108.24	–	–	128.99	–	104.31	–	101.79	–
	Mangodara	–	108.24	–	–	128.99	–	104.70	–	96.60	–
	Sindou	–	110.87	–	–	84.23	–	122.62	–	105.03	–
Centre	Baskuy	–	85.81	–	–	122.30	–	106.78	–	105.47	–
	Bogodogo	–	87.60	–	–	108.45	–	91.47	–	91.75	–
	Boulmiougou	–	85.81	–	–	122.30	–	107.47	–	105.49	–
	Nongr-Massom	–	77.72	–	–	112.06	–	96.52	–	108.16	–
	Sig-Nonghin	–	85.81	–	–	100.92	–	109.01	–	124.20	–
Centre-Est	Bittou	–	82.26	–	85.49	–	–	114.57	–	99.69	–
	Garango	–	82.26	–	85.49	–	–	112.78	–	103.22	–
	Koupéla	–	83.42	–	78.64	–	–	122.91	–	108.84	–
	Ouargaye	–	101.13	–	–	120.32	–	124.61	–	105.62	–
	Pouytenga	–	83.42	–	78.64	–	–	110.25	–	100.07	–
	Tenkodogo	–	82.26	–	85.49	–	–	120.69	–	105.37	–
	Zabré	–	82.88	–	–	109.71	–	138.99	–	110.33	–
Centre-Nord	Barsalogo	–	95.88	–	–	–	–	115.13	–	101.57	–
	Boulsa	–	93.27	–	–	94.98	–	102.44	–	103.53	–
	Kaya	–	87.11	–	–	98.89	–	103.07	–	101.76	–
	Koungoussi	–	107.81	–	–	–	–	110.58	–	105.65	–
Centre-Ouest	Koudougou	–	96.32	–	–	119.28	–	90.07	–	97.41	–
	Léo	–	90.06	–	–	111.11	–	90.62	–	103.37	–
	Nanoro	–	94.77	–	–	134.96	–	101.19	–	103.07	–
	Réo	–	99.08	–	–	–	–	92.94	–	101.82	–
	Sapouy	–	79.65	–	–	109.24	–	81.15	–	96.11	–
Centre-Sud	Kombissiri	–	92.42	–	–	96.99	–	94.01	–	107.13	–
	Manga	–	91.68	–	–	80.10	–	82.61	–	100.19	–
	Pô	–	93.95	–	–	96.69	–	97.41	–	102.99	–
	Saponé	–	104.76	–	–	111.15	–	88.43	–	104.06	–
Est	Bogandé	–	81.17	–	91.35	0	–	88.92	–	104.03	–
	Diapaga	–	82.92	–	–	91.86	–	105.63	–	98.55	–
	Fada	–	81.17	–	–	99.72	–	106.98	–	99.50	–
	Gayeri	–	100.12	–	–	93.78	–	101.50	–	109.42	–
	Manni	–	81.17	–	91.35	–	–	105.32	–	98.37	–
	Pama	–	94.04	–	–	89.09	–	105.64	–	106.22	–
Hauts Bassins	Dafra	–	86.14	–	–	109.64	–	95.11	–	104.14	–
	Dandé	–	106.23	–	–	108.26	–	129.81	–	97.39	–
	Dô	–	89.05	–	–	111.40	–	109.45	–	98.99	–
	Houndé	–	93.45	–	–	128.60	–	131.59	–	99.39	–
	Orodara	–	104.42	–	–	112.03	–	91.11	–	90.95	–
	Lena	–	86.14	–	–	109.64	–	103.17	–	107.2	–
	Karangasso Vigué	–	86.14	–	–	109.64	–	92.04	–	95.08	–
Nord	Gourcy	101.96	–	93.73	–	–	93.56	–	92.51	–	97.68
	Ouahigouya	87.42	–	96.10	–	–	92.17	–	94.32	–	98.89
	Seguenega	81.33	–	95.29	–	–	97.99	–	100.43	–	97.99
	Titao	99.27	–	100.66	–	–	92.62	–	95.59	–	102.14
	Yako	99.27	–	91.53	–	–	106.26	–	100.57	–	99.85
Plateau Central	Boussé	–	100.96	–	–	–	–	104.28	–	101.85	–
	Ziniaré	–	81.05	–	–	100.94	–	102.89	–	102.94	–
	Zorgho	–	83.73	–	–	100.66	–	108.72	–	98.62	–
Sahel	Djibo	81.23	–	86.65	–	–	92.46	–	89.69	–	96.83
	Dori	98.68	–	92.03	–	–	88.64	–	89.54	–	98.82
	Gorom	83.27	–	81.17	–	–	91.95	–	91.33	–	96.42
	Sebba	87.92	–	90.83	–	–	93.59	–	86.29	–	95.61
Sud-Ouest	Batie	117.67	–	104.49	–	–	109.22	–	89.70	–	102.05
	Dano	108.96	–	96.06	–	–	94.19	–	83.05	–	97.06
	Diebougou	72.74	–	76.25	–	–	91.29	–	92.18	–	97.74
	Gaoua	94.92	–	120.68	–	–	91.96	–	87.13	–	87.31

^a^ Calculated for each round of mass administration implemented by dividing the number of treatments distributed in the district – as reported by health workers – by the projected number of children aged 7–11 years present in the district. Projected numbers of children were based on the 2006 national census data.

Data source: Unpublished records of the National Programme for the Control of Schistosomiasis and the National Integrated Programme for the Control of Neglected Tropical Diseases, reproduced with the permission of the Ministry of Health of Burkina Faso.

In each round of praziquantel administration, trained health workers treated children of school age either in schools or – for the children who were not attending any school – in communities.^13^ A dose pole was used to measure children’s height and determine the required dose.^13^

### Baseline data

For our analyses, we used baseline data that were collected for the national programme for schistosomiasis control. These data were collected from 16 randomly selected primary schools in the hyper-endemic zone, in 2004 – before the first mass administrations of praziquantel.^15^^,^^17^ Stool and urine samples were collected from about 100 randomly selected children aged 7–14 years – half of them girls – at each of the 16 schools and checked for the eggs of *S. mansoni* and *S. haematobium*, respectively.

### Impact surveys

In 2008, the national Ministry of Health designated 22 sentinel sites for the monitoring and evaluation of the schistosomiasis programme in Burkina Faso: three in Hauts Bassins, two each in Boucle du Mouhoun, Centre-Est, Centre-Nord, Centre-Ouest, Centre-Sud, Est, Nord, Sahel and Sud-Ouest and one in Cascades. These sites, all of which were schools, were purposefully selected across 11 of the country’s 13 health regions to give a fairly even geographical distribution across the country (Fig. 1). Cross-sectional surveys in each sentinel site were done in 2008 and 2013. In each of these surveys, stool and urine samples were collected and examined for schistosome eggs. Each survey covered 160 school children aged 7–11 years – i.e. 16 boys and 16 girls from each of classes 1–5.

### Parasitological examination

One urine sample and one stool sample from each child were collected in separate containers with unique identification numbers, and sent to a laboratory for examination on the day of their collection. Urine samples were filtered through a nylon filter (pore size 12 μm; Merck Millipore, Billerica, United States of America) and the number of eggs counted under a microscope. For specimens of less than 10 ml, the volumes were measured before filtration and the number of eggs per 10 ml calculated. Intensity of S. haematobium infection was expressed as the number of eggs per 10 ml of urine examined.

The Kato–Katz method was used to check stool samples for *S. mansoni* eggs. On the day that the sample had been collected, duplicate slides were prepared from each sample and examined. Eggs were counted and intensity of infection was expressed as the number of eggs per gram of faeces.

### Data analysis

The data collected in 2013 were entered into spreadsheets and double checked by biomedical technicians. As we could not access the full data set from the 2008 assessment, we compared the data collected in 2013 with a descriptive summary of the data collected in 2008^8^ and the data collected in the 2004 baseline survey.^15^ Prevalence and intensities of infection – and their corresponding 95% confidence intervals (CI) –were calculated using SPSS version 19 (IBM, Armonk, USA). When calculating the overall values for prevalence and intensity of infection across the 11 regions with sentinel sites, the samples were adjusted with weighting according to the proportion of the national population represented by each regional population in 2013 – as projected from the results of the 2006 census. The complex-samples module of the SPSS package was used – with regions as the strata and schools as clusters – to take account of the clustering of the investigated school children. In general, our comparisons of the intensity of infection were based on the arithmetic mean egg counts for all subjects. Children were considered to have heavy S. haematobium infections if they had at least 50 eggs per 10 ml of urine.^18^ Children with more than 399 eggs per gram of faeces were considered to have heavy S. mansoni infections.^18^ Prevalence and intensities were compared using *χ^2^* and Kruskal–Wallis tests, respectively. The geographical coordinates of each sentinel site, as determined in Google Maps (Google, Mountain View, USA), were used to plot the site’s approximate position on national maps drawn in ArcMap version 10 (ESRI, Redlands, USA). Costs of the mass praziquantel administration were estimated using financial data collected in 2013 and 2014 (G Liebowitz, Helen Keller International, unpublished data, 2015). Before 2013, the relevant financial data were either incomplete or unavailable.

## Results

### Situation in 2013

Fig. 1 and Table 2 summarize the prevalence of the *S. haematobium* and *S. mansoni* infections observed among the 3514 school children – 1748 boys and 1766 girls – aged 7–11 years who provided stool and urine samples at the 22 sentinel sites. Table 2 also summarizes the mean egg counts. Although the adjusted overall prevalence of *S. haematobium* infection was 8.76%, the prevalence of such infection ranged from 0.0% (0/160) to 56.3% (90/160) according to sentinel site (median: 2.5%). The children from Centre-Est, Est and Sahel had significantly higher prevalence of *S. haematobium* infection than the children from the other eight regions (*P* < 0.001). After adjustment for the sex distribution of the national population, the proportions of the boys (9.90%) and girls (7.65%) found infected with *S. haematobium* were similar (*P* > 0.05).

**Table 2:** Prevalence and intensity of schistosome infection among children aged 7–11 years, Burkina Faso, 2013

Schistosome, region	No. of children investigated	No. infected	Prevalence of infection, % (95% CI)	No. heavily infected^a^	Prevalence of heavy infection, % (95% CI)	Mean egg count^b^ (95% CI)
***Schistosoma haematobium***						
Boucle du Mouhoun	320	20	6.25 (4.08–9.46)	11	3.44 (1.93–6.05)	9.86 (2.84–16.88)
Cascades	160	0	0.00 (0.00–2.34)	0	0.00	–
Centre-Est	320	110	34.38 (29.38–39.74)	28	8.75 (6.12–12.36)	20.08 (10.39–29.77)
Centre-Nord	320	16	5.00 (3.10–7.97)	3	0.94 (0.32–2.72)	1.72 (0.62–2.83)
Centre-Ouest	320	4	1.25 (0.49–3.17)	1	0.31 (0.06–1.75)	0.68 (0.00–1.84)
Centre-Sud	320	7	2.19 (1.06–4.45)	4	1.25 (0.49–3.17)	1.37 (0.15–2.59)
Est	314	57	18.15 (14.28–22.79)	10	3.18 (1.74–5.76)	6.60 (3.22–9.98)
Hauts Bassins	480	0	0.00 (0.00–0.79)	0	0.00	–
Nord	320	5	1.56 (0.67–3.60)	1	0.31 (0.06–1.75)	1.11 (0.00–3.08)
Sahel	320	67	20.94 (16.84–25.73)	37	11.56 (8.51–15.53)	24.47 (14.33–34.60)
Sud-Ouest	320	1	0.31 (0.06–1.75)	0	0.00	0.10 (0.00–0.30)
***Schistosoma mansoni***						
Boucle du Mouhoun	320	0	0.00	0	0.00	–
Cascades	160	0	0.00	0	0.00	–
Centre-Est	320	0	0.00	0	0.00	–
Centre-Nord	320	0	0.00	0	0.00	–
Centre-Ouest	320	0	0.00	0	0.00	–
Centre-Sud	320	1	0.31 (0.06–1.75)	0	0.00	0.15 (0.00–0.45)
Est	314	0	0.00	0	0.00	–
Hauts Bassins	480	42	8.75 (6.54–11.62)	1	0.21 (0.04–1.17)	7.7 (4.18–11.22)
Nord	320	0	0.00	0	0.00	–
Sahel	320	0	0.00	0	0.00	–
Sud-Ouest	320	0	0.00	0	0.00	–
All investigated	3514	43	1.15 (0.84–1.55)^c^	1	0.03 (0.01–0.16)^c^	1.00 (0.26–1.75)^c^

CI: confidence interval.

^a^ Children were considered to have heavy S. haematobium infections if they had at least 50 eggs per 10 ml of urine and to have heavy S. mansoni infections if they had more than 399 eggs per gram of faeces.

^b^ Calculated for all of the children investigated, irrespective of their infection status. Counts of *S. haematobium* and *S. mansoni* eggs were per 10 ml of urine and per gram of faeces, respectively.

^c^ This value was weighted according to the proportion of the national population represented by each regional population in 2013 – as projected from the results of the 2006 census.

The adjusted arithmetic mean intensity of *S. haematobium* infection – among all children investigated – was 6.0 eggs per 10 ml urine. The mean egg counts for the children from Boucle du Mouhoun, Centre-Est and Sahel were significantly higher than those for the children from the other eight regions (*P* < 0.001). Boys were generally more heavily infected than girls ( *P* = 0.013). The adjusted overall prevalence of heavy *S. haematobium* infection was 2.82%. The Centre-Est (8.75%; 28/320) and Sahel regions (11.56%; 37/320) had the highest percentages of children infected. In six of the regions included in the assessment, less than 1% of the children investigated had *S. haematobium* infection. Overall, 3.83% of the boys investigated and 1.8% of the girls were found heavily infected with *S. haematobium* (*P* > 0.05).

*S. mansoni* was only detected in the Hauts Bassins region – with a prevalence of 8.75% (42/480) and an arithmetic mean egg count of 7.7 per gram of faeces – and the Centre-Sud region – with a prevalence of 0.31% (1/320) and an arithmetic mean egg count of 0.15 per gram of faeces.

### Data for 2004 and 2008

The prevalence of *S. haematobium* recorded in the 22 sentinel sites during the national survey in 2008 was, in general, markedly higher than that recorded in 2013 (Fig. 1).

Table 3 shows the baseline data collected in 2004 from the Boucle du Mouhoun, Nord, Sahel and Sud-Ouest^15^ and the corresponding data, from the same four regions, from the assessment in 2013. As these two sets of data were collected in different sites and different numbers of sites – and the exact locations of the sites surveyed in 2004 could not be determined – we made no direct statistical comparisons between the two data sets and could not produce a map of the baseline data to match our other figures. However, the data in Table 3 indicate that, between 2004 and 2013, there were large reductions in both the prevalence and intensity of *S. haematobium* infection in the Boucle du Mouhoun, Nord, Sahel and Sud-Ouest regions.

**Table 3:** Changes in prevalence and intensity of *Schistosoma haematobium* infection among children aged 7–11 years from four regions, Burkina Faso, 2004 and 2013

Variable	No. of children investigated		Prevalence				Mean egg count		
			% (95% CI)		Reduction, %		Eggs/10 ml urine (95% CI)^b^		Reduction, %
	2004^a^	2013	2004^a^	2013			2004^a^	2013	
**Region**									
Boucle du Mouhoun	413	320	58.6 (53.8–63.3)	6.25 (4.08–9.46)	89.3		106.7 (86.0–127.5)	9.86 (0–22.95)	90.8
Nord	417	320	61.2 (56.5–65.8)	1.56 (0.67–3.60)	97.5		91.0 (67.3–114.6)	1.11 (0–3.09)	98.8
Sahel	412	320	84.2 (80.7–87.7)	20.94 (16.84–25.73)	75.1		126.9 (99.3–154.4)	24.47 (11.77–37.16)	80.7
Sud-Ouest	402	320	18.4 (14.6–22.2)	0.31 (0.06–1.75)	98.3		39.4 (22.8–56.1)	0.10 (0–0.30)	99.7
All four	1644	1280	55.8 (53.4–58.2)	7.50 (6.18–9.08)^c^	86.6		91.3 (80.0–102.7)	9.40 (4.03–14.76)^c^	89.7
**Sex**									
Male	936	637	59.8 (56.7–63.0)	8.50 (6.57–10.92)^c^	85.8		111.8 (95.6–128.1)	5.13 (2.50–7.76)^c^	95.4
Female	708	643	50.6 (46.9–54.2)	6.53 (4.87–8.70)^c^	87.1		64.2 (49.1–79.3)	13.74 (3.24–24.25)^c^	78.6

CI: confidence interval.

^a^ Baseline data.^15^^,^^17^

^b^ Calculated for all of the children investigated, irrespective of their infection status.

^c^ This value was weighted according to the proportion of the total combined population of the four regions represented by each regional population in 2013 – as projected from the results of the 2006 census.

### Drug distribution costs

At the beginning of the national programme for schistosomiasis control, the cost of a round of mass treatment with praziquantel was estimated to be 0.32 United States dollars (US$) per child treated.^13^ Helen Keller International’s financial accounts indicated that the costs of schistosomiasis treatment – including the costs of drug transportation and distribution, supervision of the distribution, training of drug distributors and social mobilization within the integrated programme for the control of neglected tropical diseases – totalled US$ 209 761.71 in 2013 and US$ 422 404.49 in 2014. These costs, which reportedly covered the treatment of 8 243 795 people – i.e. 4 068 082 in 2013 and 4 175 713 in 2014 – indicate that the mean cost of a round of mass treatment with praziquantel in 2013–2014 was about US$ 0.08 per person treated.

## Discussion

After a decade of preventive chemotherapy, progress has been made in Burkina Faso in the control of schistosomiasis – at a modest cost. In the 2013 assessment, the prevalence of schistosome infection among school children was found to be below 5% in five of the 11 included regions – and below 10% in eight of the regions. In the two regions not included in the 2013 national assessment – i.e. Centre and Plateau Central – the Ministry of Health also found the prevalence of *S. haematobium* infection to be below 5% in 2013.^19^ In 2013, therefore, recorded prevalence of *S. haematobium* infection remained high – i.e. above 18% – in only three regions: Centre-Est, Est and Sahel. In addition, the heavy *S. haematobium* infections that are associated with most of the morbidity of urogenital schistosomiasis were only rarely detected – i.e. in less than 1% of the children checked in eight regions included in the 2013 national assessment or the smaller ministry of health study.^19^ According to the criteria of the World Health Organization (WHO),^20^ by 2013, these eight regions had eliminated urogenital schistosomiasis as a public health problem. By the same year, another three regions – i.e. those in which 1–5% of children surveyed were found to have heavy *S. haematobium* infections – had reached the target of controlling the morbidity caused by such schistosomiasis.^20^

Despite the generally encouraging trends revealed by our analyses, there were some causes for concern. For example, the Centre-Est and Sahel regions appeared to have failed to control urogenital schistosomiasis by 2013. At one Centre-Est sentinel site, the prevalence of *S. haematobium* infection was much higher in 2013 (56.3%) than in 2008 (14.4%). Similarly, in a Hauts Bassins sentinel site, the prevalence of *S. mansoni* infection recorded in 2013 (26.3%) was higher than that recorded in 2008 (17.9%). At several sites in the Centre-Est and Est regions, the prevalence of *S. haematobium* infection recorded in 2013 was similar to that recorded in 2008. There are at least three possible reasons for an increase or persistence in the prevalence of infection. First, the frequency of treatment may be inadequate, especially in areas with particularly high levels of infection and transmission. Second, even though the overall coverage of mass administration may appear adequate, focal treatment coverage may not be satisfactory. Third, there may be particular social or environmental factors that are supporting focal transmission despite the benefits of the preventive chemotherapy. The results of ongoing research in the Centre-Est region may help to explain the local persistence of schistosomiasis foci.

After studying the results of the 2013 assessment and the relevant WHO recommendations,^20^^,^^21^ the managers of the national programme against neglected tropical diseases have recently reviewed the progress achieved, set objectives for the next phase of the programme and increased treatment frequency in some areas. The objectives are now to use mass drug administrations: (i) biennially, to interrupt the transmission of *S. haematobium* and *S. mansoni* in the Cascades, Centre, Centre-Nord, Centre-Ouest, Centre-Sud, Nord, Plateau Central and Sud-Ouest regions; (ii) annually, to control schistosome-related morbidity or eliminate schistosomiasis as a public health problem in the Boucle du Mouhoun, Est, Hauts Bassins and Sahel regions; and (iii) biannually, to control schistosome-related morbidity or eliminate schistosomiasis as a public health problem in the Centre-Est region.^8^ At the same time, schistosomiasis surveys are to be extended to non-sentinel areas to check that the trends seen at the sentinel sites are nationally representative and identify any foci of transmission that have not been recognized previously.

Although it has long been known that regular treatment with praziquantel can prevent both the severe and milder morbidity associated with schistosomiasis,^6^^,^^17^^,^^22^^,^^23^ there is an indication in the data from Burkina Faso that it may also lead to the elimination of schistosomiasis in certain transmission settings. Burkina Faso is a land-locked country that is usually divided into three climate zones: the north-Sudanese in the south, the sub-Sahelian in the centre and the Sahelian in the north – with annual rainfall of 900–1200, 600–900 and 400–600 mm, respectively.^24^ In much of the country, water is a scarce resource. Surface water consists of two main rivers that carry water all year around – i.e. the Mouhoun and Nakambe rivers – several perennial water reservoirs and some seasonal water bodies.^25^ In previous studies in Burkina Faso and Niger, the prevalence of *S. haematobium* infection was found to be reduced by one round of mass drug administration and then to remain low for another 2–3 years in the absence of further chemotherapy.^15^^,^^26^^,^^27^ In these countries, which have relatively little perennial surface water, the risks of re-infection after mass drug administration with high coverage are relatively low. This may explain why repeated biennial mass drug administration in Burkina Faso appears to have effectively eliminated schistosomiasis as a public health problem in at least eight regions.

Apart from increasing treatment frequency where necessary, other complementary public health interventions may need to be considered in Burkina Faso – particularly in the persistent foci with high prevalence of infection. WHO has recommended comprehensive measures for eliminating neglected tropical diseases^28^ and complementary measures that could be introduced in a phased approach to the control of schistosomiasis^20^ – e.g. health education, improved sanitation and access to clean water, environmental snail control and focal use of molluscicides.^29^^–^^32^ In Burkina Faso, snail management and operational research on molluscicide use are needed. Closer collaboration between the integrated programme for the control of neglected tropical diseases and the education and communications sectors are needed to support behavioural change communications to change water-contact behaviour and minimize the risk of infection. The integrated programme and the water, sanitation and hygiene sectors also need to work together to reduce transmission.
